# Neuroimmunological Effect of Vitamin D on Neuropsychiatric Long COVID Syndrome: A Review

**DOI:** 10.3390/nu15173802

**Published:** 2023-08-30

**Authors:** Ting-Bin Chen, Ching-Mao Chang, Cheng-Chia Yang, I-Ju Tsai, Cheng-Yu Wei, Hao-Wen Yang, Chun-Pai Yang

**Affiliations:** 1 Department of Neurology, Neurological Institute, Taichung Veterans General Hospital, Taichung 407219, Taiwan; sophiebeen@gmail.com; 2 Center for Traditional Medicine, Taipei Veterans General Hospital, Taipei 11217, Taiwan; magicbjp@gmail.com; 3 Faculty of Medicine, College of Medicine, National Yang Ming Chiao Tung University, Taipei 11217, Taiwan; 4 Institute of Traditional Medicine, National Yang Ming Chiao Tung University, Taipei 11217, Taiwan; 5 Department of Healthcare Administration, Asia University, Taichung 41354, Taiwan; chengchia@asia.edu.tw; 6 Department of Medical Research, Kuang Tien General Hospital, Taichung 433, Taiwan; hunch0815@hotmail.com; 7 Department of Exercise and Health Promotion, College of Kinesiology and Health, Chinese Culture University, Taipei 11114, Taiwan; 8 Department of Neurology, Chang Bing Show Chwan Memorial Hospital, Changhua 50544, Taiwan; 9 Department of Family Medicine, Kuang Tien General Hospital, Taichung 433, Taiwan; 10 Department of Neurology, Kuang Tien General Hospital, Taichung 433, Taiwan; 11 Department of Nutrition, HungKuang University, Taichung 433, Taiwan; 12 Ph.D. Program in Translational Medicine, National Chung Hsing University, Taichung 402, Taiwan

**Keywords:** long COVID, vitamin D, neuroimmunological effect

## Abstract

Severe acute respiratory syndrome coronavirus 2 (SARS-CoV-2) is the causative agent of the coronavirus disease 2019 (COVID-19). COVID-19 is now recognized as a multiorgan disease with a broad spectrum of manifestations. A substantial proportion of individuals who have recovered from COVID-19 are experiencing persistent, prolonged, and often incapacitating sequelae, collectively referred to as long COVID. To date, definitive diagnostic criteria for long COVID diagnosis remain elusive. An emerging public health threat is neuropsychiatric long COVID, encompassing a broad range of manifestations, such as sleep disturbance, anxiety, depression, brain fog, and fatigue. Although the precise mechanisms underlying the neuropsychiatric complications of long COVID are presently not fully elucidated, neural cytolytic effects, neuroinflammation, cerebral microvascular compromise, breakdown of the blood–brain barrier (BBB), thrombosis, hypoxia, neurotransmitter dysregulation, and provoked neurodegeneration are pathophysiologically linked to long-term neuropsychiatric consequences, in addition to systemic hyperinflammation and maladaptation of the renin–angiotensin–aldosterone system. Vitamin D, a fat-soluble secosteroid, is a potent immunomodulatory hormone with potential beneficial effects on anti-inflammatory responses, neuroprotection, monoamine neurotransmission, BBB integrity, vasculometabolic functions, gut microbiota, and telomere stability in different phases of SARS-CoV-2 infection, acting through both genomic and nongenomic pathways. Here, we provide an up-to-date review of the potential mechanisms and pathophysiology of neuropsychiatric long COVID syndrome and the plausible neurological contributions of vitamin D in mitigating the effects of long COVID.

## 1. Introduction

Severe acute respiratory syndrome coronavirus 2 (SARS-CoV-2), a type of betacoronavirus, is the causative agent of coronavirus disease 2019 (COVID-19). It predominantly leads to respiratory-related symptoms, with the majority of infected people being either asymptomatic or experiencing self-limiting disease progression. The remaining cases manifest as either mild or severe [1]. SARS-CoV-2 enters host cells by binding its spike protein to the angiotensin-converting enzyme 2 (ACE2), a cell surface protein widely expressed in the respiratory and gastrointestinal tissues. This interaction leads to direct virus-mediated cytopathological damage, disruption of the renin–aldosterone–angiotensin system (RAAS) as a result of ACE2 pathway maladaptation, endothelial damage, platelet activation, hypercoagulability, and an excessive immune response, which drive hyperinflammation and hypercytokinemia, ultimately leading to organ injury through aberrant innate and acquired immune responses [2,3,4,5,6,7].

Following the initial critical stage, the inflammatory response gradually subsides, the damaged organs gradually recover, and some of the damaged organs progress to the fibrosis and chronic stage. This chronic phase includes conditions such as chronic critical illness, persistent inflammation, immunosuppression, and catabolism syndrome [3]. Numerous patients across different age groups continue to experience incapacitating symptoms similar to the multiorgan damage observed in the acute phase of infection, regardless of whether their initial infection was severe, mild, or even asymptomatic. These symptoms persist over an extended period after recovery [8,9,10]. Long COVID encompasses a constellation of new, returning, or ongoing symptoms lasting more than 4 weeks after acute infection with SARS-CoV-2, which cannot be explained by an alternative diagnosis. The National Institutes of Health introduced the concept of postacute sequelae of SARS-CoV-2 infection, which involves a broad spectrum of symptoms persisting well beyond the recovery phase of the initial COVID-19 stage, encompassing various clinical syndromes, including postintensive care syndrome, chronic fatigue syndrome, postacute COVID-19 syndrome (i.e., signs and symptoms persisting between 4 and 12 weeks from the date of onset), and post-COVID-19 syndrome (i.e., signs and symptoms persisting beyond 12 weeks from date of onset) [11,12,13].

A wide range of persistent multisystem dysfunctions constituting the long COVID syndrome have been identified across studies [14,15]. Accurately distinguishing the sequelae of acute COVID-19 from the symptoms derived from the hospitalization itself, deterioration of preexisting chronic diseases, adverse effects of medications, or the emergence of new conditions triggered by the initial COVID-19 infection is clinically important. Moreover, differentiating between lingering symptoms of the acute disease and newly arising symptoms subsequent to the resolution of the acute phase can identify potential drivers of diseases or dysfunctions. Furthermore, as the number of COVID-19 hospitalized patients continues to decline, the neurological manifestations of long COVID predominantly affect the younger demographic of nonhospitalized patients, thereby causing a substantial socioeconomic impact [16].

The potential pathophysiological mechanisms underlying long COVID syndrome include pathological inflammation (e.g., occult viral persistence and systemic or tissue-localized immune dysregulation), induced autoreactive immunity (e.g., antibodies against the ACE2 receptor, hypocretin receptor, interferon, neutrophils, connective tissues, cyclic citrullinated peptides, and cell nuclei), reactivation of human herpesviruses (e.g., Epstein–Barr virus), changes in the gut microbiome, endothelial dysfunction, and activation of coagulation processes [12,17,18,19,20,21]. Long COVID syndrome is a complex heterogeneous multisystem disorder presenting a wide spectrum of clinical features (both pulmonary and extrapulmonary). These manifestations include respiratory, cardiovascular, hematologic, and neuropsychiatric symptoms either alone or in combination [22,23]. Common clinical symptoms of this syndrome are sleep disturbances, fatigue, dyspnea, brain fogginess, memory impairment, headaches, loss of smell or taste, cough, depression, anxiety, palpitations, dizziness, myalgia, and arthralgia [10,24,25,26].

Vitamin D consists of a group of structurally related secosteroids, including cholecalciferol, ergocalciferol, 25-hydroxyvitamin D (25(OH)D, calcidiol), and 1,25-dihydroxyvitamin D (1,25(OH)2D, calcitriol). Vitamin D plays a key role not only in bone metabolism but also in the body’s defense against contagious agents such as bacteria, viruses, parasites, and fungi [27]. This immunomodulatory function is mediated by the vitamin D receptor (VDR) and the activating enzyme 25-hydroxyvitamin D-1alpha-hydroxylase (CYP27B1) in immune cells through the modulation of proinflammatory cytokines (e.g., IL-6, TNF-alpha, and interferon-gamma), Th1 lymphocyte response, microbicidal activity of phagocytes, and release of antimicrobial peptides. Vitamin D is believed to counteract or prevent detrimental hyperimmune states resulting from acute COVID-19 and as severe consequences of COVID-19, such as acute respiratory distress syndrome, microvascular thrombosis, and cytokine storm, with hyperinflammation as an underlying basis for all of these conditions [28,29]. The overall pathophysiology of neuropsychiatric long COVID syndrome is complex and encompasses persistent systemic inflammation and cytokine storm. In addition to its role in immune and inflammatory modulation during acute COVID-19, vitamin D is implicated in the downregulation of the RAAS and improvement of disrupted glucose homoeostasis, the coagulation cascade, and cardiovascular health [28,29,30,31,32,33]. Notably, vitamin D deficiency or insufficiency is widespread among COVID-19 patients and is associated with increased mortality and adverse outcomes in acute COVID-19. This is because vitamin D deficiency contributes to the state of hyperinflammation and may exacerbate preexisting metabolic and cardiovascular diseases [34,35,36,37,38,39]. Consequently, vitamin D has been proposed as one of the crucial components in the treatment of acute COVID-19 infection [40,41,42,43].

Although vitamin D deficiency or insufficiency is prevalent in COVID-19 survivors [44,45], studies investigating its association with neuropsychiatric long COVID syndrome are limited. The primary objective of the present study was to provide a comprehensive overview of the promising neuroimmunological mechanisms of vitamin D for long-term neuropsychiatric consequences and to provide an updated review of this topic.

## 2. Neuropsychiatric Symptoms of Long COVID

Neurological and psychiatric symptoms are observed in >35% of individuals with acute COVID-19 [46,47,48,49]. These individuals present various manifestations involving the central nervous system (CNS), peripheral nervous system (PNS), neuromuscular junctions, and skeletal muscles. Initial symptoms include sleep disturbances, headaches, chemosensory impairment, and myalgia [47,50,51]. The multifactorial and complicated pathogenic mechanisms of SARS-CoV-2, which exhibit neurotropic properties, contribute to the wide spectrum of neuropsychiatric manifestations during acute COVID-19. These can arise either through direct cytolytic effects or as a result of secondary hyperinflammatory reactions (indirect effects). The virus can invade the nervous system through leukocyte migration across the BBB, infection of the vascular endothelium, retrograde axonal transport through the cranial nerves, and transsynaptic viral spread, thereby causing direct cytopathic injuries in neurons and glial cells and neurotransmitter imbalance [52,53]. Moreover, SARS-CoV-2 infection may functionally divert the bioenergetic capacity of infected cells to support viral replication, thereby disrupting mitochondrial homeostasis, impairing mitophagy, and compromising neuronal function [54,55,56]. Once the virus gains entry into the nervous system, it binds to ACE2, which is highly expressed in the substantia nigra, thalamus, choroid plexus, olfactory bulb, ventricles, middle temporal gyrus, posterior cingulate cortex, and brainstem. ACE2 is present in both neuronal and nonneuronal cell types (astrocytes, oligodendrocytes, pericytes, and endothelial cells) [57,58,59]. This interaction of the virus with ACE2 promotes neuroinflammation, hypercoagulation, microhemorrhages, disruption of the BBB, endothelitis or microvasculitis, generation of reactive species, and hypoxia [53,60,61]. Neuropsychiatric impairment may occur through two main pathways: (1) brain damage resulting from cytolytic effects, macro- and micro-hypoxic/ischemic injuries, neuroinflammation, and BBB dysfunction and (2) systemic toxic-metabolic dysfunction secondary to diffuse alveolar and interstitial inflammatory exudation, multiorgan dysfunction, sepsis/septic shock, systemic hyperinflammation, hypercoagulation, thromboembolism, endocrine or electrolyte imbalance, and effects of pharmacological agents [53]. Numerous studies on the neuropsychiatric consequences of COVID-19 have demonstrated that certain significant changes in the cerebrospinal fluid and plasma biomarkers and brain images may serve as diagnostic and prognostic tools during the acute infection phase [62,63,64,65].

Neuropsychiatric long COVID syndrome encompasses various presentations of compromised integrative neurological functions that regulate key cognitive and affective processes in the CNS in COVID-19 survivors [66]. Of these survivors, an estimated 31–69% experience long COVID symptoms after initial recovery from SARS-CoV-2 infection [67]. Among the long COVID symptoms, fatigue (37–47%), sleep disorders (e.g., insomnia and excessive sleepiness) (31%), myalgia (25%), headaches (15–18%), chemosensory impairment (7–14%), cognitive dysfunction (22–32%) (e.g., brain fog, memory impairment, and attention disorder), anxiety (23%), depression (17%), and autonomic dysfunction are common neuropsychiatric manifestations, some of which substantially increase in prevalence over time and exert adverse effects on patients’ quality of life and ability to work [68,69,70,71]. As new evidence continually emerges, the spectrum of clinical characteristics of neuropsychiatric long COVID syndrome continues to broaden. In contrast to the overlapping pathogenetic mechanisms implicated in the neurological manifestations of acute COVID-19, the underlying biological causes of neuropsychiatric long COVID sequelae remain poorly delineated to date. The proposed pathophysiological mechanisms are mainly inferred on the basis of the pathophysiology of acute COVID-19. Persistent symptoms may result from a combination of neurobiological and psychological factors.

The overall pathophysiology of neuropsychiatric long COVID syndrome is complex and involves a combination of factors such as persistent systemic inflammation/cytokine storm [70], SARS-CoV-2 neurotropism [60], prolonged neuroinflammation [70,72], BBB disruption [70,73,74], generation of autoantibodies [70], microvasculitis [73,74], prolonged endothelial and platelet activation [73,74], enhanced thrombin generation [73], hypoxia [73,74], dysregulation of neurotransmitters [72], amyloid aggregation [75], tau phosphorylation [76], protein misfolding [75], and neuronal death [75], in addition to prolonged systemic inflammation and maladaptative changes in the RAAS [70,77,78]. Moreover, chronic activation of the extended autonomic system (including neuroendocrine and neuroimmune systems) and the hypothalamus–pituitary–adrenal axis, physical deconditioning, psychological challenges (e.g., posttraumatic stress and fear of infecting others or stigmatization), and social and financial effects contribute, in part, to the persistence of psychiatric problems in the long term [72,79]. Furthermore, specific gut microbiome profiles are associated with the persistence of long COVID, suggesting that dysbiosis and disruptions in the brain–gut axis might play a vital role in the development of long COVID [80]. Moreover, persistent SARS-CoV-2 infection can lead to the following: (1) mitochondrial dysfunction, causing increased oxidative stress and ultimately resulting in the loss of mitochondrial integrity and cell death; (2) binding of viral proteins to mitochondrial complexes, disrupting mitochondrial function and causing the immune cells to continually overreact; and (3) epigenetic alterations (e.g., reduced telomere length and changes in DNA methylation) [81,82]. Furthermore, female sex, respiratory symptoms at the onset of infection, severity of acute COVID-19, admission to the intensive care unit, presence of comorbidities, history of mental disease, and elevated levels of inflammatory markers are associated with an increased risk of chronic neuropsychiatric manifestations [83,84].

Processes such as hippocampal and cortical atrophy, hypoxic changes, and small vessel disease are relevant neurological processes that emerge as secondary to neuroinflammation, oxidative stress, BBB breakdown, disrupted proteostasis, and autophagic dysfunction during COVID-19 [60,75,85,86,87,88,89]. Notably, an increasing body of research has indicated that SARS-CoV-2 infection can initiate or accelerate neurodegeneration in surviving patients through the activation of the inflammasome, mislocalization of the tau protein from the axons to soma, hyperphosphorylation of tau, and impaired clearance and pathological accumulation of amyloid and tau proteins, ultimately leading to neuronal death [90]. The consequences of these neuropathological processes may manifest over the long term through changes in brain images. These changes include findings such as white matter lesions evident on magnetic resonance imaging (MRI), disruptions in the microstructural and functional integrity of the brain observed on diffusion tensor imaging, hyperactivation in specific brain regions such as the piriform cortex and anterior cingulate gyrus observed on functional MRI, hypometabolism in the frontal cortex detected through fluorodeoxyglucose positron emission tomography, reduced cerebral perfusion detected through single photon emission computed tomography, widespread cerebral hypoperfusion observed on arterial spin labeling MRI, and alterations in cortical excitability and activity of neurotransmitters. Furthermore, blood biomarkers indicative of neural injury and neuroinflammation contribute to understanding the scope of these neurological changes [91,92,93,94,95,96,97].

The scope and complexity of neuropsychiatric sequelae resulting from long COVID may cause substantial morbidity and impose a substantial socioeconomic burden on patients, families, the healthcare system, and society at large. Because the prevalence of neuropsychiatric symptoms appears to increase over time, a holistic and multidisciplinary approach to their management becomes imperative. This approach can encompass various components such as nutritional support, personalized and adapted physical and cardiopulmonary rehabilitation, psychological support, and cognitive training. Such an approach is crucial to promptly identify potentially life-limiting complications, mitigate the catastrophic consequences of long COVID, and enhance both physical and mental well-being, ultimately improving the overall quality of life of COVID-19 survivors.

## 3. Vitamin D and Its Neuroimmunological Roles

Vitamin D has gained increased attention in diverse areas of medical research because of its multifaceted role extending beyond bone mineralization and calcium homeostasis. The vitamin D endocrine system provides a wide range of skeletal and extra-skeletal functions. Low vitamin D levels are believed to contribute to the development and progress of autoimmunity, infectious diseases, diabetes mellitus, cardiometabolic disorders, obesity, cancer, gastrointestinal diseases, intestinal dysbiosis, stroke, dementia, and depression [98,99,100,101,102,103]. Vitamin D, a fat-soluble neuroactive prohormone, is increasingly recognized as not only a marker of overall health but also a necessary neurosteroid and immunomodulator, exerting pleiotropic effects on the neurological system. Vitamin D is mainly synthesized by the skin (~80%) from 7-dihydrocholesterol following ultraviolet B exposure. Vitamin D becomes biologically active in humans after undergoing sequential hydroxylation in the liver to 25(OH)D, which is catalyzed by CYP2R1 and CYP27A1, and then in the kidney to the fully active metabolite 1,25(OH)2D, which is catalyzed by CYP27B1 [104]. In a smaller proportion (~20%), vitamin D can also be obtained through dietary intake. Vitamin D metabolites circulate within the bloodstream, typically bound to the vitamin D binding protein (DBP). Among these metabolites, 25(OH)D is vital and is clinically assessed as an indicator of an individual’s vitamin D level [105]. The concentration of 1,25(OH)2D in the blood is strictly regulated through a feedback mechanism involving 1,25(OH)2D itself, parathyroid hormone, calcium, fibroblast growth factor 23, and various cytokines [106].

Vitamin D and its metabolites have the ability to traverse the BBB, and they exert their effects through both genomic and nongenomic pathways, contributing to their multidirectional extra-skeletal benefits. Genomic responses are typically observable over several hours to days, whereas nongenomic responses trigger the rapid activation of various signaling cascades within 1–2 min and 15–45 min [107]. These actions stimulate the transcription and expression of numerous target genes (regulating 3% of the human genome; i.e., >900 genes) participating in numerous cellular processes related to cardiometabolic functions, immune responses, neuroprotection, antioxidation, maintenance of mitochondrial integrity, and cellular proliferation and differentiation. These actions are facilitated by the direct binding of vitamin D metabolites to a nuclear receptor (i.e., VDR), which forms a heterodimer with the retinoid-X-receptor [99,104,108]. VDR and the enzymes involved in vitamin D hydroxylation are widely expressed in various immune cells and brain tissues, including dendritic cells, macrophages, lymphocytes, cerebral endothelial cells, pericytes, neurons, astrocytes, and microglia distributed throughout the CNS, including areas such as the cortex, limbic regions (amygdale, hippocampus, and hypothalamus), deep gray matter (thalamus, basal ganglia, and nucleus accumbens), and the substantia nigra. This widespread distribution highlights their critical roles in both immunological and neurological functions [99,104,108,109,110]. Furthermore, vitamin D metabolites can elicit rapid nongenomic actions in a paracrine and autocrine manner by modulating the expression of genes through a membrane-associated rapid-response steroid-binding protein (i.e., protein disulfide isomerase A3, PDIA3). This mechanism not only contributes to the classical genomic pathway but also enables cross-talk with various signaling pathways, modulating inflammation, apoptosis, oxidative stress, and phosphorylation of cellular proteins, as well as neuron excitability and other electrophysiological phenomena [111,112]. Notably, the expression of PDIA3 in the brain, particularly in regions critical to neurocognitive function, is orders of magnitude greater than its expression in the liver and kidneys [113]. The molecular mechanisms of vitamin D and its metabolites in genomic and nongenomic pathways across large brain areas provide a foundation for understanding their crucial neuropsychiatric functions. This understanding is supported by accumulating in vivo and in vitro evidence indicating that the neurochemical and physiological actions of vitamin D metabolites involve effects on neuroimmunomodulation, neuronal differentiation, neuronal maturation, cellular proliferation, mitochondrial respiratory chain, redox balance, oxidative phosphorylation, calcium signaling/homeostasis, neurotrophism, cerebral angiogenesis, neural circuitry, neuroprotection, neurogenesis, synaptogenesis, synaptic plasticity, neurotransmission/neurotransmitter regulation, and amyloid clearance [99,100,114,115,116]. The robust biological effects of vitamin D on CNS and PNS cells contribute to the protection of the CNS from inflammation at the cellular level (including the BBB and glia). This protection is achieved through multiple mechanisms including the secretion of cytokines and growth factors, cell signaling, response to oxidative stress, regulation of BBB integrity, and trafficking, as well as the support of myelination, axonal homogeneity in peripheral nerves, and neuronal-cell differentiation [117,118].

The level of vitamin D is influenced by various factors, including the extent of sunlight exposure (related to factors such as latitude, season, sedentary indoor lifestyles, sunscreen use, sun avoidance, clothing habits, and home confinement/quarantine), air pollution, personal factors (such as age, sex, ethnicity, body mass index, skin pigmentation, and medication use), underlying medical comorbidities (such as renal and hepatic insufficiency), and genetic factors (nucleotidic polymorphisms of the genes *DBP*, *NADSYN1/DHCR7*, *CYP2R1*, *CYP24A1*, and *VDR*) [108,119,120,121,122,123]. However, modifiable environmental or personal factors, instead of genetic variants, were found to be the main determinants of vitamin D levels [122]. Severe vitamin D deficiency (serum 25(OH)D concentration <30 nmol/L) requires correction. However, most guidelines recommend maintaining serum 25(OH)D concentrations of >50 nmol/L for optimal skeletal health in older populations [124]. However, whether this range is suitable for preserving CNS integrity and neuropsychiatric function remains unclear because both low and excessively high serum vitamin D levels are associated with neurocognitive deficits in an inverse U-shaped manner [113]. Accumulating evidence from clinical, epidemiological, and basic science studies indicates that vitamin D insufficiency is widespread across all age groups and even in healthy populations, irrespective of the geographical location or seasonal variation. Moreover, vitamin D deficiency has been demonstrated to be mechanistically and clinically linked to the pathogenesis of various neurological, psychiatric, and autoimmune disorders, such as cerebrovascular diseases, Alzheimer’s disease, Parkinson’s disease, multiple sclerosis, depression, and schizophrenia [98,101,103,104,108,124,125,126,127].

The inconsistent results in clinical interventional studies on the role of vitamin D supplementation in the development or progress of autoimmune and neuropsychiatric diseases can be attributed, in part, to various methodological factors, such as differences in patient cohorts, varied doses of vitamin D administered, different study designs, and bioanalytical determination methods used [108,124,128]. Although vitamin D deficiency is a common risk factor, the origin of these disorders is complex. Whether vitamin D deficiency is a causal factor influencing the etiology of these illnesses or is a consequence of them, coupled with lifestyle changes and reduced sun exposure, remains debatable.

Apart from its recognized benefits for overall systemic health, such as its anti-infection properties, its supporting role in mitochondrial function, cardiometabolic benefits, immunomodulatory effects, and anti-inflammatory effects, vitamin D exerts effects on immunomodulation or anti-inflammation, neuroprotection, regulation of monoamine neurotransmission, preservation of BBB integrity, vasculometabolic functions, modulation of gut microbiota, and enhancement of telomere stability. These multifaceted roles highlight the importance of vitamin D in maintaining normal brain functioning as well as preventing and managing neuropsychiatric, neuroinflammatory, and neurodegenerative disorders.

### 3.1. Immunomodulation and Anti-Inflammation

Vitamin D is a well-known active regulator of both innate and adaptive immunity and plays a crucial role in safeguarding the nervous system against invasive pathogens [129]. Regarding innate immunity, vitamin D enhances the synthesis of antimicrobial peptides such as defensin beta 2 and cathelicidin in macrophages and monocytes. Additionally, it promotes chemotaxis, autophagy, and phagolysosomal fusion in macrophages and natural killer cells. Furthermore, vitamin D reinforces gut microbiota homeostasis and enhances the physical barrier function of intestinal epithelial cells [103,130]. With respect to adaptive immunity, vitamin D promotes an anti-inflammatory state by regulating T cells, B cells, and antigen-presenting cells (dendritic cells and macrophages) through the suppression of the activation of Th1 cells, inhibition of the production of proinflammatory cytokines, and modulation of the activities of Th2 cells, T regulatory cells, and Th17 cells [101,103,130]. Overall, as a tolerogenic, anti-inflammatory cytokine, vitamin D not only aids the immune system in dampening excessive or chronic reactions but also facilitates the rapid and effective elimination of pathogens. The immune regulatory effects induced by vitamin D in peripheral tissues may also extend protection to the CNS by locally reinforcing the BBB and reducing glial activation in the brain parenchyma.

Vitamin D exerts anti-inflammatory effects on human microglia by inhibiting the production of proinflammatory cytokines and facilitating differentiation of M2 macrophages and the upregulation of the anti-inflammatory Toll-like receptor, thus inhibiting inflammation-driven neural injury [123]. Moreover, it serves as a potent antioxidant during inflammatory processes. It achieves this by enhancing defective autophagy and mitochondrial function through the inhibition of the mTOR pathway. Vitamin D also reduces oxidative stress and inflammatory responses by activating FoxO-dependent antioxidant pathways, inhibiting NF-κB signaling, promoting reactive oxygen species-scavenging enzymes, downregulating NADPH oxidase, and upregulating Nrf2 [123]. Vitamin D can be regarded as a pivotal molecule for promoting cell survival. This is achieved by synchronizing calcium oscillatory signaling in cells, regulating autophagy or apoptosis, and critically regulating the inflammasome during stress responses. Additionally, it promotes protein homeostasis and longevity through stress response pathway genes such as *skn-1*, *ire-1*, and *xbp-1* [131,132]. Furthermore, it downregulates the expression of adhesion molecules such as intercellular adhesion molecule-1 and vascular cell adhesion molecule-1 in endothelial cells. This modulation of adhesion molecules influences the BBB by controlling the migration of immune cells to the CNS [117,133]. Collectively, neuroinflammation and oxidative stress play fundamental roles in neural injury, BBB breakdown, and the progression of neurodegenerative processes. These processes are key factors underlying a wide range of neurological, psychiatric, and cardiovascular diseases, such as Alzheimer’s disease, Parkinson’s disease, multiple sclerosis, amyotrophic lateral sclerosis, and depression [101,134,135,136], rendering these processes rational targets for new therapies even in individuals with sufficient vitamin D levels.

### 3.2. Neuroprotection

Vitamin D regulates neuronal differentiation and maturation, enhances neuronal survival, and prevents neurotoxicity through the following mechanisms: (1) promotion of the synthesis of essential neurotrophic factors, such as nerve growth factor, glial cell line-derived neurotrophic factor, brain-derived neurotrophic factor, and neurotrophins; (2) modulation of neuronal calcium homeostasis through the downregulation of L-type voltage-sensitive calcium channels and synthesis of calcium-binding proteins, such as calbindin, parvalbumin, and calretinin; (3) reduction of inducible nitric oxide synthase and increases in glutathione, superoxide dismutase, and arginase-1, thereby protecting against mitochondrial dysfunction; and (4) enhancement of the synthesis of other neurosteroids such as estrogen and progesterone [112,117,137]. Additionally, it affects synaptic or neuronal plasticity, axiogenesis or axonal growth, neuronal myelination, and the maintenance of the cytoskeleton and cell transport of organelles through the regulation of numerous proteins, such as drebrin, growth-associated protein-43, connexin 43, synapsin-1, neurofilament, tubulin, actin, microtubule-associated protein-2, glial fibrillary acidic protein, creatine kinase b, kinesin, and dynactin [138,139]. Vitamin D may have beneficial effects for neurodegenerative processes associated with Alzheimer’s disease. It can help attenuate the hyperphosphorylation of the tau protein, counteract neuronal death, mitigate amyloid-induced cytotoxicity, and regulate amyloid homeostasis. It achieves this by (1) enhancing the clearance of amyloid plaques through increased phagocytosis by macrophages and facilitating brain-to-blood efflux across the BBB, mediated by low-density lipoprotein receptor related protein-1, and (2) reducing the amyloid production and increasing its degradation by factors such as nicastrin, neprilysin, and secretases [140,141,142,143,144,145]. The consequences of long-term deficiency or inefficient utilization of vitamin D can disrupt these neuroprotective mechanisms, potentially rendering neurons more susceptible to aging and neurodegeneration.

### 3.3. Monoamine Neurotransmission

Vitamin D actively regulates the genetic processes responsible for the synthesis of acetylcholine, dopamine, serotonin, and gamma-aminobutyric acid, all of which modulate a broad spectrum of neuropyschological functions, including mood, cognition, learning, memory, reward processing, and sleep [146,147].

### 3.4. BBB Integrity

The functional and structural integrity of the BBB is crucial for maintaining the homeostasis of the brain microenvironment. This semipermeable membrane is essential for regulating the influx and efflux of biological substances at the brain–blood interface, which is fundamental for supporting the brain’s metabolic activity and neuronal function. Vitamin D has potential benefits for neurovascular coupling and BBB function. Within the neurovascular unit, various enzymes including CYP27A1, CYP27B1, and CYP24A1 are expressed in astrocytes, endothelial cells, microglia, and oligodendrocytes, and CYP27B1 is abundantly expressed in neurons [148]. The VDR is primarily expressed in astrocytes, whereas PDIA3 is abundantly expressed in all cerebral cell types [148]. Brain pericytes lack the expression of CYP27B1 and are therefore unable to synthesize 1,25(OH)D. This observation suggests that pericytes rely on paracrine or circulating 1,25(OH)D to maintain neurovascular function [148]. Brain microvascular endothelial cells have a notable ability to transform cholecalciferol into 25(OH)D. They also express VDR and PDIA3, contributing to the regulation of BBB transporters (e.g., permeability-glycoprotein, multidrug resistance-associated protein 1, and breast cancer resistance protein) and tight-junction proteins (e.g., occludin, claudin-5, and zonula occludens-1) [117,148]. Thus, vitamin D may play a vital role in counteracting a deleterious cascade of injury processes and functional deficits that result from compromised BBB integrity.

### 3.5. Vasculometabolic Functions

Vitamin D exerts various systemic cardiovascular effects through VDR-mediated mechanisms in cardiomyocytes and vascular endothelial cells as well as through the regulation of the RAAS, adiposity, energy expenditure, and pancreatic cell activity [149]. In the genomic pathway of cerebral endothelial cells, the expression of genes encoding stromal cell-derived factor 1α, vascular endothelial growth factor, and nitric oxide synthase is upregulated, contributing to vasodilation and anti-inflammation. Insulin-like growth factor 1 is involved in the neuroprotection of axons and dendrites and thrombolysis through the activation of plasminogen. The tight-junction proteins of the BBB ensure microvascular structural integrity and microcirculatory function. Nerve growth factors are also influenced by this genomic pathway [150]. Collectively, these beneficial genomic effects on vascular endothelial cells result in reduction of thrombogenicity, reduction of vasoconstriction, inhibition of oxidative/nitrative stress and atherogenesis, enhancement of endothelial repair, decrease in foam cell formation, and improvement of vascular relaxation and dilatation [151]. Additionally, vitamin D plays a role in regulating matrix homeostasis through the modulation of specific matrix metalloproteinases and tissue inhibitors of metalloproteinases, all of which are critical in major cerebrovascular diseases, including atherosclerosis, arteriosclerosis, and stroke [123]. By combining its vasculometabolic actions with the potent systemic and neural immunomodulatory and antioxidant effects, vitamin D can positively modulate cerebral vascular homeostasis and BBB function and prevent vascular dysfunction and tissue injury as a result of systemic and local (neural) inflammation.

### 3.6. Gut Microbiota

The VDR plays a role in regulating the gut microbiome by suppressing inflammation, maintaining barrier function, promoting microbial homeostasis, and reducing insulin resistance [152,153,154]. More than 90% of the body’s serotonin is synthesized in the gut, predominantly by enterochromaffin cells. Disruption of the intestinal microbiota triggered by inflammatory processes has been shown to directly compromise the synthesis of this monoamine [155]. Vitamin D supplementation has been demonstrated to promote an increase in serum serotonin levels in individuals with depression as well as in serum dopamine levels in children with attention deficit/hyperactivity disorder [155]. Studies have suggested that vitamin D may serve as a key regulator in the gut–brain axis, modulating gut microbiota and alleviating psychiatric symptoms such as depression and anxiety [155,156]. Consequently, vitamin D deficiency leads to dysbiosis, a probable reason for the increased vulnerability to inflammation-mediated illnesses.

### 3.7. Telomere Stability

Vitamin D has been demonstrated to be involved in telomere biology and genomic stability through various pathways. It has the potential to preserve telomere length, inhibit cellular aging, and mitigate telomere shortening through anti-inflammatory and anti-cell proliferation mechanisms [157]. Significantly low levels of telomerase activity lead to the development of shortened telomeres, which in turn triggers cell cycle arrest, ultimately leading to cell senescence and apoptosis [157].

## 4. Vitamin D and Its Potential Benefits for Neuropsychiatric Long COVID Syndrome

The long-term impact of COVID-19 on survivors, including those with mild or no symptoms, highlights the need to identify the pathophysiological mechanisms that contribute to the development of long COVID syndrome. A large body of evidence supports that vitamin D is required for maintaining normal immune function, combating pathogens, and preventing autoimmune diseases. Until now, the association between vitamin D and numerous neurocognitive and neuropsychiatric illnesses, such as neurodegenerative diseases, stroke, autism, schizophrenia, depression, and attention deficit hyperactivity disorder, has been extensively explored [137,158,159]. The profound effect of vitamin D on neuroinflammatory and neurodegenerative processes within the CNS through both genomic and nongenomic pathways has been demonstrated. The complex mechanisms of its neuroprotective effects involve both VDR-mediated and nongenomic effects on neurotrophin expression, mitigation of L-type calcium channel expression, mitigation of oxidative stress, excitotoxicity, apoptosis, and promotion of neuroprotective and antiaging processes through interference with multiple prosurvival signaling pathways. The ability of vitamin D to attenuate neuroinflammation is noteworthy, as evidenced by the decreased expression and release of proinflammatory cytokines and nitric oxide. In addition, through critical mechanisms such as alleviation of defective autophagy and enhancement of mitochondrial function, immunomodulation, anti-inflammation responses, neuroprotection, monoamine neurotransmission, maintenance of BBB integrity, vasculometabolic function, regulation of the gut microbiota or gut–brain axis, and maintenance of telomere stability, vitamin D potentially protects the brain from various pathophysiologies implicated in neuropsychiatric long COVID syndrome. These include persistent neuroinflammation, BBB breakdown, autoimmunity, thrombus formation, disrupted neurotransmission, and neurodegeneration, as well as prolonged systemic inflammation and RAAS dysregulation. The conflicting current clinical data regarding the effects of vitamin D intervention may be attributed to inadequate dosing, variability in trial designs that hinders direct comparisons, diverse routes of administrations employed, and insufficient consideration of the age- and sex-related effects of vitamin D pharmacology. Nevertheless, vitamin D has emerged as a strong candidate for modulating the wide range of neuropsychiatric symptoms and pathophysiologies encountered in different stages of the long COVID syndrome.

## 5. Future Perspective

Gaining a deep understanding of the pathophysiology of neuropsychiatric long COVID is essential to effectively evaluate and manage the consequences of this condition. Although robust and conclusive evidence derived from well-designed randomized trials demonstrating the efficacy of regular daily vitamin D supplementation for mitigating the full spectrum of neuropsychiatric symptoms in individuals who have recuperated from COVID-19 is lacking, its overall favorable attributes across various domains (e.g., neurological, psychiatric, cardiovascular, and immune) might support the consideration of vitamin D as a potential health supplement or an adjuvant treatment. This might be relevant for maintaining both physical and mental well-being and managing neuropsychiatric symptoms in both the acute and long COVID phases.

Long COVID syndrome encompasses a diverse range of symptoms, with wide variation among individuals. These symptoms often result from complex interactions between various physiological and immunological processes, making it improbable for a single intervention, such as vitamin D supplementation, to be universally effective for the treatment of all aspects of this complex syndrome. Therefore, prior to strongly advocating for the application of vitamin D as a stand-alone remedy or in conjunction with other therapeutic agents for managing neuropsychiatric symptoms associated with long COVID syndrome, several pivotal aspects must be elucidated. Critical considerations include determining the optimal dosage, treatment duration, and ideal formulation or delivery method to ensure the optimal absorption and efficacy of vitamin D. Striking a balance between treatment benefits and potential risks, identifying specific target symptoms, identifying the patient group that might benefit the most from this approach, and establishing methods for assessing therapeutic responses are all challenging tasks and are still under investigation.

## 6. Conclusions

Long COVID continues to persist despite vaccination and booster doses, with highly debilitating neuropsychiatric manifestations. This comprehensive review described the pathophysiological mechanisms by which vitamin D may confer resistance against long COVID based on the existing body of evidence. The literature strongly supports the capacity of vitamin D to modulate both immune cells and neural cells through both genomic and nongenomic pathways. Beyond its systemic health benefits (e.g., anti-infection effects, metabolic benefits, immune regulation, and anti-inflammatory effects), the favorable effects of vitamin D on neural immunomodulation/anti-inflammation, neuroprotection, monoamine neurotransmission, BBB integrity, and vasculometabolic functions indicates its indispensable role in preserving normal brain function as well as in preventing and managing neuropsychiatric, neuroinflammatory, and neurodegenerative processes in long COVID. Although these therapeutic roles of vitamin D in combating long COVID are promising, this review may serve as a foundation for further exploration of its effect on long COVID. Undoubtedly, additional epidemiological, experimental, and randomized control trials are warranted to substantiate and translate these proposed effects in the context of long COVID.

## Data Availability

Not applicable.
